# The complete mitochondrial genome sequence and gene organization of *Cryodraco antarcticus* (Perciformes, Channichthyidae) with phylogenetic consideration

**DOI:** 10.1080/23802359.2020.1718023

**Published:** 2020-02-04

**Authors:** Ping Cao, Wei Song, Hongliang Huang, Lingzhi Li, Keji Jiang, Xuezhong Chen

**Affiliations:** a Key Laboratory of Oceanic and Polar Fisheries, Ministry of Agriculture, East China Sea Fisheries Research Institute, Chinese Academy of Fishery Sciences, Shanghai, China;; b College of Fisheries and Life Sciences, Shanghai Ocean University, Shanghai, China

**Keywords:** *Cryodraco antarcticus*, mitochondrial genome, gene structure, phylogenetic tree

## Abstract

The complete mitochondrial genome DNA sequence of *Cryodraco antarcticus* was 17,857 bp in size. It consists of 13 protein-coding genes, 2 ribosomal RNAs, 22 transfer RNAs, and one control region. Among 22 tRNA genes, 8 tRNAs were encoded on the L-strand. The overall base composition of the genome is 26.45% for A, 25.96% for T, 29.78% for C, and 17.81% for G. The phylogenetic tree suggested *C. antarcticus* was genetically closest to some species in family Channichthyidae. This study could provide valuable information for further studies on population structure, conservation genetics and molecular evolution of *C. antarcticus.*

*Cryodraco antarcticus*, a member of Channichthyidae, broadly distributes in the Antarctic Ocean. It is benthic and carnivorous, and its maximum length of body is 39.30 cm. Furthermore, it always perches on 300–800 m underwater (Kock and Jones 2002). Mitochondrial DNA plays a significant part in the studies of population genetics, phylogenetic, and evolution (Avise et al. 1984; Zhong et al. 2013; Xia et al. 2015). So far, there was no introduction about the complete mitochondrial genome of *C. antarcticus*. The study is important for the further research on genetics and evolution of *C. antarcticus*.

The specimen of *C. antarcticus* whose Specimen Accession number is Esfri-2016LD-3-SW was collected from Antarctic (61°13′30″S, 63°36′48″W). It was stored in the East China Sea Fisheries Research Institute, Chinese Academy of Fishery Science. Genomic DNA was extracted from muscle tissue using Animal Genomic DNA Extraction Kit (TIANGEN, Beijing, China) according to the manufacturer’s recommended protocol. In the present study, the full length of complete mitochondrial DNA of *C. antarcticus* has been sequenced by the Roche 454 Genome Sequencer FLX System. The total length was 17,857 bp (GenBank accession No. MK941847). The base composition of its mitogenome is 26.45% for A, 25.96% for T, 29.78% for C, and 17.81% for G. The overall A + T content of the mitochondrial genome is 52.41%. The complete mitogenomic sequence obtained includes 13 protein-coding genes, 2 ribosomal RNAs, 22 transfer RNAs, and 1 control region. 28 of these 37 genes were encoded on the heavy strand, and nine were encoded on the light strand just as in other teleosts (Song et al. 2016). The overall length of the protein-coding genes is 10,405 bp. Three kinds of start codons (ATG, ATC, and GTG) and four types of stop codons (TAA, TAG, TA–, T––) were identified in 13 protein-coding genes. And four genes (ND1, COX1, ATP6, ND4L) ended with TAA, eight genes (ND2, COX2, COX3, ND3, ND4, ND5, CYTB, ND6) had incomplete stop codons TA– or T––, one gene (ATP8) ended with TAG. The length of control region (D-loop) is 2027 bp, and its overall nucleotide composition is 28.76% for A, 25.95% for C, 18.21% for G, and 27.08% for T.

To assess its phylogeny and evolution, the phylogenetic tree was constructed with significant bootstrap supports based on the Neighbour-joining method in MEGA 5.1 (Figure 1). *Larimichthys crocea*, *Larimichthys polyactis*, and *Collichthys lucidus* were used as an out-group. The NJ tree showed that *C. antarcticus* clustered with some species in family Channichthyidae, like *Chaenodraco wilsoni* and *Chaenocephalus aceratus*, then together with other species in family Notothenioidei forming a big branch. This study will be important to the genetic conservation and the phylogenetic classification of *C. antarcticus*.

**Figure 1.**
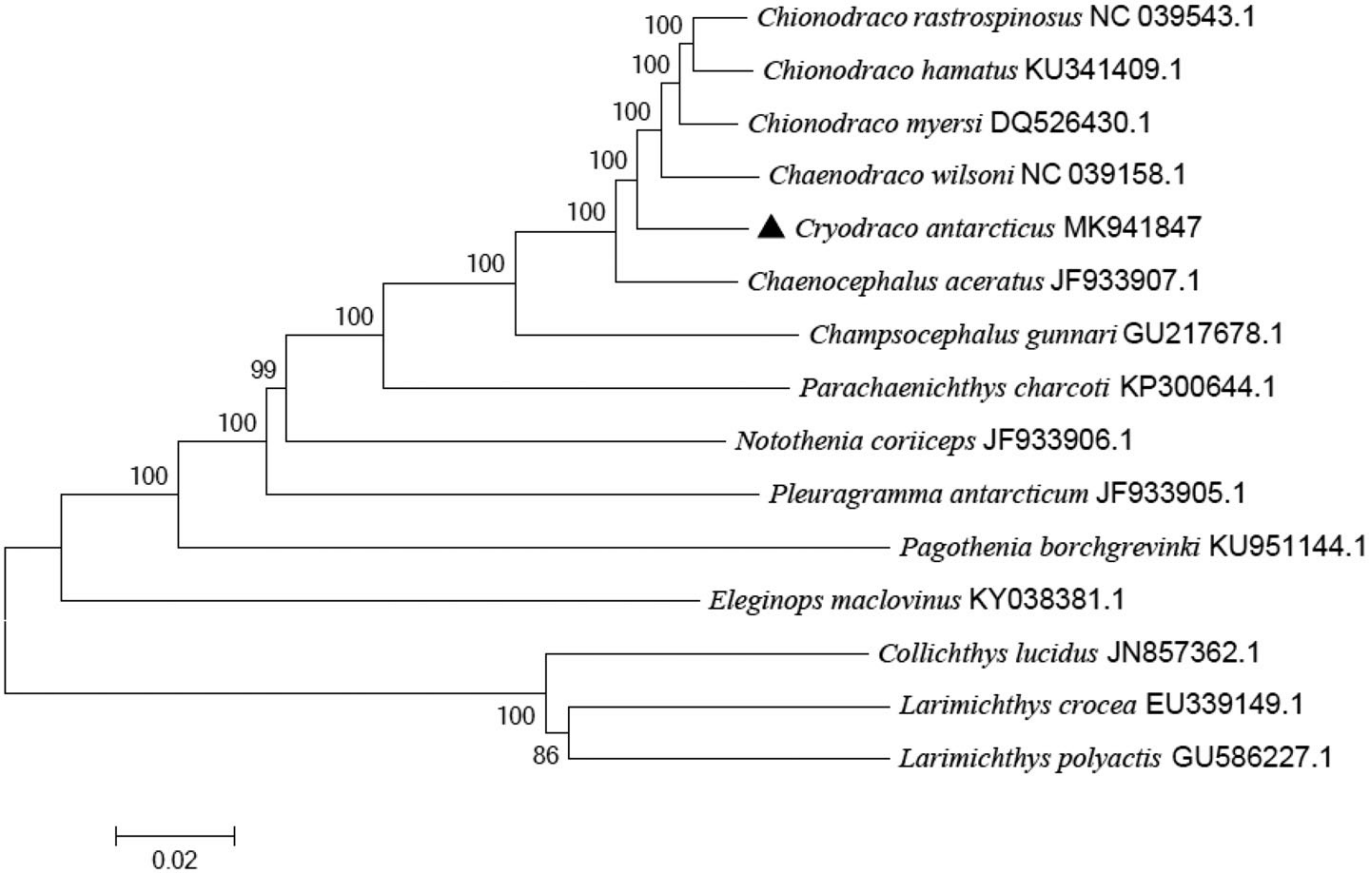
The phylogenetic tree based on complete mtDNA sequences using the neighbour-joining method in MEGA 5.1. *Cryodraco antarcticus* was highlighted with a black triangle.
